# Improving inference for aerial surveys of bears: The importance of assumptions and the cost of unnecessary complexity

**DOI:** 10.1002/ece3.2912

**Published:** 2017-05-25

**Authors:** Joshua H. Schmidt, Tammy L. Wilson, William L. Thompson, Joel H. Reynolds

**Affiliations:** ^1^ Central Alaska NetworkU.S. National Park ServiceFairbanksAKUSA; ^2^ Southwest Alaska NetworkU.S. National Park ServiceAnchorageAKUSA; ^3^ Department of Natural Resource ManagementSouth Dakota State UniversityBrookingsSDUSA; ^4^ U.S. Fish and Wildlife ServiceHadleyMAUSA; ^5^ Western Alaska Landscape Conservation CooperativeAnchorageAKUSA; ^6^ Present address: U.S. National Park ServiceKingstonRIUSA; ^7^ Present address: U.S. National Park ServiceAnchorageAKUSA

**Keywords:** apparent bias, availability bias, brown bear, detection probability, distance sampling, informative prior, mark–recapture distance sampling, open *N*‐mixture model, perception bias, precision, *Ursus arctos*

## Abstract

Obtaining useful estimates of wildlife abundance or density requires thoughtful attention to potential sources of bias and precision, and it is widely understood that addressing incomplete detection is critical to appropriate inference. When the underlying assumptions of sampling approaches are violated, both increased bias and reduced precision of the population estimator may result. Bear (*Ursus* spp.) populations can be difficult to sample and are often monitored using mark‐recapture distance sampling (MRDS) methods, although obtaining adequate sample sizes can be cost prohibitive. With the goal of improving inference, we examined the underlying methodological assumptions and estimator efficiency of three datasets collected under an MRDS protocol designed specifically for bears. We analyzed these data using MRDS, conventional distance sampling (CDS), and open‐distance sampling approaches to evaluate the apparent bias‐precision tradeoff relative to the assumptions inherent under each approach. We also evaluated the incorporation of informative priors on detection parameters within a Bayesian context. We found that the CDS estimator had low apparent bias and was more efficient than the more complex MRDS estimator. When combined with informative priors on the detection process, precision was increased by >50% compared to the MRDS approach with little apparent bias. In addition, open‐distance sampling models revealed a serious violation of the assumption that all bears were available to be sampled. Inference is directly related to the underlying assumptions of the survey design and the analytical tools employed. We show that for aerial surveys of bears, avoidance of unnecessary model complexity, use of prior information, and the application of open population models can be used to greatly improve estimator performance and simplify field protocols. Although we focused on distance sampling‐based aerial surveys for bears, the general concepts we addressed apply to a variety of wildlife survey contexts.

## 1 Introduction

Obtaining useful estimates of wildlife abundance or density requires thoughtful attention to potential sources of bias and precision affecting the population estimator (Williams, Nichols, & Conroy, 2002). For instance, properly accounting for bias associated with incomplete detection of individuals has been consistently difficult to address when surveying wildlife populations. This difficulty has led to the development and implementation of a wide range of survey tools such as distance sampling, mark–recapture, occupancy, and double observer approaches (e.g., Buckland et al., 2001; MacKenzie et al., 2002; Nichols et al., 2000; Rosenstock, Anderson, Giesen, Leukering, & Carter, 2002; Williams et al., 2002). Each of these techniques addresses specific sources of incomplete detection, although the detection process consists of four individual detection components: *p*_s_ (the probability that the home range of an animal overlaps the sampled space), *p*_p_ (the probability that the animal is present within the survey area during the survey period), *p*_a_ (the probability that the animal is available to be detected given that it is present within the sampled area), and *p*_d_ (the probability that the animal is detected given that it is present and available; see Nichols, Thomas, & Conn, 2009; Schmidt, McIntyre, & MacCluskie, 2013). Many commonly implemented field sampling techniques focus on correcting for *p*_d_ < 1.0, and although *p*_s_ is typically addressed through design (Nichols et al., 2009), *p*_a_ and *p*_p_ are less often estimated explicitly or dealt with through design considerations. Instead, inference is often based on the assumption that all individuals are present and available to be detected, although temporary emigration (Kendall, Nichols, & Hines, 1997) or incomplete availability (Schwarz & Arnason, 1996; Wilson, Schmidt, Thompson, & Phillips, 2014) can cause bias relative to the population of interest. Avoidance of these sources of bias is important if results are to be interpretable, and hence useful for addressing research or management questions.

Situations where *p*_p_ and *p*_a_ approach 1.0 certainly exist, although this requires careful assessment within each survey context. For example, if the species of interest has a small home range and exhibits slow movement rates relative to the survey period and study area size, assuming *p*_p_ ≈ 1.0 might be reasonable. Conversely, highly mobile species (e.g., passerine birds) may move to areas outside of a particular sample unit on any particular visit (Schmidt et al., 2013). Similarly, highly visible species in open habitats may meet the assumption of *p*_a_ ≈ 1.0 (e.g., Dall's sheep; Schmidt, Rattenbury, Lawler, & MacCluskie, 2012), whereas in other cases, behavior or individual characteristics could be expected to cause *p*_a_ ≈ 0 for some individuals during part of the survey period (e.g., diving cetaceans; Laake, Calambokidis, Osmek, & Rugh, 1997). When it is unreasonable to assume that all individuals are present and available, *p*_p_ and *p*_a_ must be addressed directly through careful attention to design and/or analysis; otherwise inference is limited to some unknown proportion of the population, despite correcting for *p*_d_ < 1.0. Although partially corrected population indices can be useful in some contexts (e.g., Johnson, 2008), inference to the entire study population is generally of primary interest to scientists and managers. When assumptions related to *p*_p_ and *p*_a_ cannot be met through design modifications (e.g., use of radiotelemetry), analytical solutions become necessary to achieve estimates that are interpretable and support comparisons across time or space.

In addition to addressing bias in the overall detection process, consideration must be given to important tradeoffs among bias, precision, and survey costs (both logistical and monetary) within the context of an estimator's total error (i.e., total error = $\sqrt{{\text{bias}}^{2}+\text{varience}}$; Reynolds, 2012). Designs employing mark–recapture methods are often preferred in wildlife work, in part because *p*_d_, *p*_p_, and *p*_a_ can be estimated directly, thereby minimizing bias. However, the costs of capturing and marking sufficient numbers of animals may not always be practical, particularly when inference over large areas is required (Pollock et al., 2002). The expense of the marking efforts can limit feasible sample sizes, reducing estimator precision and increasing total error relative to an approach that does not attempt to discern the different sources of detection bias. In many contexts, methods based on detections of unmarked individuals (e.g., occupancy surveys; MacKenzie et al., 2006; distance sampling; Buckland et al., 2001, 2004) are simpler and cheaper to implement than other approaches, leading to larger sample sizes, fewer parameters to be estimated, and potentially smaller total error. For example, conventional distance sampling (CDS) only requires a single visit and relies on the assumption that *p*_a_ = 1.0 (although see Amundson, Royle, & Handel, 2014), whereas multiple visit approaches (e.g., occupancy surveys) can be used to incorporate *p*_a_ < 1 directly. A primary consideration when assessing the appropriateness of any particular survey approach is the relative balance it provides between bias and precision for feasible sample sizes and, specifically, the magnitude of bias it may be susceptible to while meeting precision objectives.

Although realized sample sizes are influenced by factors such as design requirements, cost, and animal density, model fitting in a Bayesian analytical framework provides another option for increasing estimator efficiency (precision) after data have been collected. Borrowing information across surveys through the use of informed priors (Gelman, Carlin, Stern, & Rubin, 2004; King, Morgan, Giminez, & Brooks, 2010; Link & Barker, 2010) provides a means for greatly increasing precision without increasing required sample sizes for “new data” (e.g., McCarthy & Masters, 2005; Schmidt & Rattenbury, 2013). Similarly, open *N*‐mixture models (Dail & Madsen, 2011) and recent extensions thereof (e.g., Kery & Royle, 2016; Schmidt, Johnson, Lindberg, & Adams, 2015; Sollmann, Gardner, Chandler, Royle, & Sillett, 2015; Zipkin et al., 2014) could be used to extract additional information from data collected on populations over time or assess assumptions regarding components of the detection process (i.e., *p*_p_ and *p*_a_; Kery & Royle, 2016). These open formulations use repeated survey data to directly estimate parameters of population dynamics (e.g., survival and recruitment) and could be used to further reduce total estimator error.

The challenges associated with designing efficient and effective abundance surveys are well illustrated by the problem of how best to monitor bear populations. Bears are notoriously difficult and expensive to monitor (Reynolds, Thompson, & Russell, 2011) because they are often secretive and solitary, occur at relatively low densities, and are difficult to detect (i.e., noncontrasting coloration). Mark‐resight techniques using radio‐marked bears and aerial surveys have been used extensively to estimate bear abundance (e.g., Miller et al., 1997), although the capture and handling requirements often limit the scale of such studies. Mark‐resight approaches based on individual characteristics have also be used successfully (Schmidt, Rattenbury, Robison, Gorn, & Shults, 2017), as have DNA mark–recapture techniques (Boulanger et al., 2008; Gardner, Royle, & Wegan, 2009; Gardner, Royle, Wegan, Rainbolt, & Curtis, 2010; Kendall et al., 2008; Solberg, Bellemain, Drageset, Taberlet, & Swenson, 2006). As a potentially cheaper alternative for estimating bear abundance at large spatial scales, double‐observer mark–recapture distance sampling (MRDS) approaches have been developed, implemented, and modified over time (Becker & Christ, 2015; Becker & Quang, 2009; Quang & Becker, 1997; Walsh et al., 2010). The MRDS approach currently represents one of the more commonly employed survey techniques for assessing bear populations at large spatial scales, particularly in remote areas. However, this approach requires large sample sizes that can be cost prohibitive, limiting utility for long‐term monitoring of bear populations (e.g., Reynolds et al., 2011).

Here, we explore the application of alternative analytical tools in the interest of cost reduction and improved inference for bear datasets collected under the MRDS approach.

We used three brown bear datasets to examine methodological and analytical assumptions, detect and reduce apparent bias, increase precision, and simplify field methods. Our specific objectives were to (1) explore the tradeoff between apparent bias and precision between the MRDS method and a simpler CDS framework for aerial bear survey data, (2) investigate the potential for improved inference through the use of informed priors on the detection process, and (3) explicitly test the assumption *p*_a_ = 1.0 through application of open‐distance sampling models.

## 2 Materials and Methods

### 2.1 Study area

**Figure 1:**
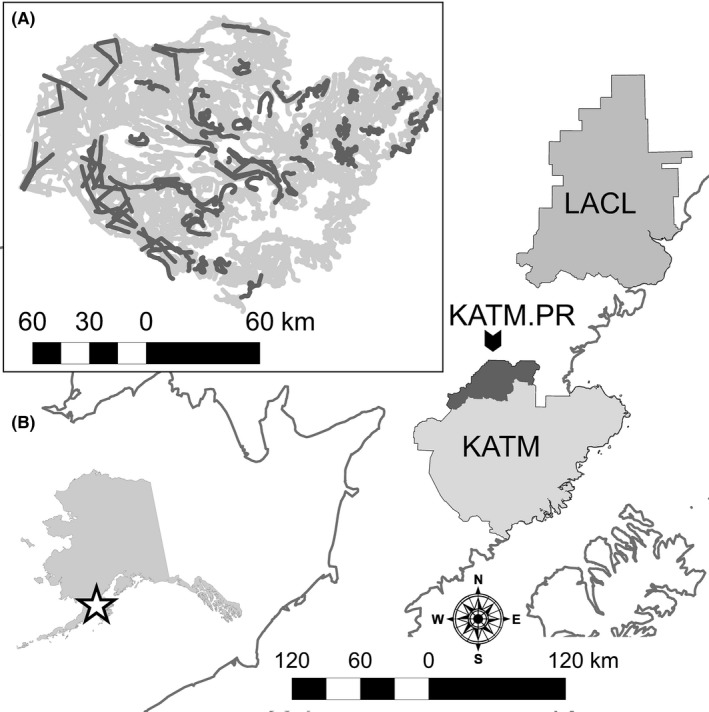
Location of the three survey units: Lake Clark National Park and Preserve (LACL), Katmai National Park and preserve (KATM), and Katmai National Preserve (KATM.PR). Map inset A depicts all of the transects surveyed in KATM (light gray). The darker gray lines depict transects that were surveyed on 18 May 2005, an example of a single day when survey transects were spatially distributed throughout the study area. The areas lacking transects represent areas not considered to be bear habitat. Map inset B shows the location of the study areas within Alaska, USA

The study area consisted of portions of Lake Clark National Park and Preserve [LACL] and Katmai National Park and Preserve [KATM] in southwestern Alaska, USA (Figure 1; Table 1). The maritime climate is characterized by cool wet summers. Winter months tend to be drier on average, but deep snow accumulates on the higher, glaciated peaks. Habitat consists of glaciated mountains, sparsely vegetated alpine plateaus, alder slopes, and forested bottomlands. Brown bears also use coastal sedge meadows and beaches. Bear densities in these coastal areas are approximately 10‐fold higher than in interior areas (Miller et al., 1997).

**Table 1:** Details of survey effort for each of the three example brown bear datasets collected in southwestern Alaska, USA

	LACL	KATM	KATM.PR
Year	2003	2004/2005	2009
Survey dates	May 18–29	May 21–30/16–26	May 18‐June 6
Study area	4,677 km^2^	18,150 km^2^	1,254 km^2^
Area surveyed	7,116 km^2^	11,939 km^2^	3,266 km^2^
Number of transects	660	639	288
Target length	20 km	25 km	15 km
Truncation distance	600 m	800 m	800 m
Number of groups detected	227	384	89
Number of groups for CDS post‐truncation	195	316	68

### 2.2 Data collection

Staff from the U.S. National Park Service and the Alaska Department of Fish and Game used an MRDS approach (Becker & Christ, 2015; Becker & Quang, 2009) to survey brown bears in LACL in 2003, KATM in 2004–2005, and in the Katmai National Preserve [KATM.PR] in 2009 (see Table 1, Figure 1). We considered these datasets to be well‐suited for our work because they were collected using a MRDS approach specifically developed for bears (Becker & Quang, 2009) and included sufficient sample sizes for analysis. The KATM survey was conducted over two consecutive spring periods to meet sample size objectives; population closure between years was assumed for the purposes of analysis. Transects were surveyed for brown bears from tandem‐seat, fixed‐wing aircraft flown at approximately 100 m above the ground. The pilot and observer independently searched for bears on the uphill side (or a random side in flat areas) of the aircraft. Detected groups were not immediately announced, allowing the other observer an opportunity to detect the group independently. To maintain observer independence, a curtain was installed in the aircraft to ensure the detection of a bear group by one observer did not alert the other observer to its presence. After a detected group had passed from the observable space, the detection was announced and the detection history (i.e., pilot only, observer only, both pilot and observer) was recorded. The location and size of the group were recorded, and the distance of the group from the line was later calculated using GIS. Further details on survey methodology can be found in Becker and Quang (2009) and Walsh et al. (2010).

### 2.3 Data analysis

In order to assess the underlying assumptions of the sampling design and analytical tools for data of this type, we used four basic approaches to analyze these three datasets: (1) MRDS following the basic approach of Becker and Christ (2015), (2) CDS following the approach of Schmidt et al. (2012), (3) CDS modified to include informed priors on the detection process (i.e., Schmidt & Rattenbury, 2013), and (4) open CDS models accounting for *p*_a_ < 1.0 (Sollmann et al., 2015). Comparing estimators with different assumptions facilitated their assessment in the context of the bias‐variance tradeoff and total estimator error. Although we did not assess absolute bias, we expected a comparison of different approaches with the well‐established MRDS approach for estimating bear abundance would provide a reasonable assessment of the “apparent bias” for each estimator. A comparison of the MRDS and CDS results provided an assessment of the apparent bias due to incomplete detection at the apex of the detection function. The addition of informed priors allowed insight into the role of sample size on estimator precision. Finally, the open‐distance approach directly assessed the assumption of *p*_a_ = 1.0 inherent in the other approaches. The results and implications based on these analyses were then used to develop broad recommendations for improving future aerial line transect surveys of bears.

#### 2.3.1 Becker‐Christ (“B‐C”) approach

Histograms of the observed distances collected under the MRDS bear survey protocol are typically “humped,” increasing to an apex at some distance from the line and then decreasing thereafter (Becker & Quang, 2009). This distribution violates the typical assumption of CDS that numbers of detections decrease monotonically with increasing distance from the transect (Buckland et al., 2001). Initially such datasets were analyzed using a gamma‐shaped detection function (Becker & Quang, 2009; Walsh et al., 2010), which can accommodate humped detection data. However, this formulation was found to violate the assumption of full independence, producing negative bias in the resulting estimators (Benson, 2010). Recently, the gamma‐function was replaced with a two‐piece normal distribution assuming point independence at the apex of the detection curve (i.e., Borchers, Laake, Southwell, & Paxton, 2006), thereby correcting for the negative bias in the original model formulation (Becker & Christ, 2015). Hereafter we refer to this analytical approach as the “B‐C approach.” Because the B‐C approach and its earlier variants have routinely been applied to datasets similar to ours, we used estimates based on it as a baseline for comparison with the other approaches in the context of apparent bias and precision.

We obtained the two piece normal source code for the B‐C approach from the original publication (Becker & Christ, 2015) and used it to estimate the number of bears in LACL, KATM, and KATM.PR. Effective search distance (ESD: how far out the observers were searching at the time the bear was detected) is a covariate thought to be necessary to account for heterogeneity that would affect the mark–recapture part of the MRDS approach (Becker & Christ, 2015). We found that both ESD and equal search‐distance categories were highly correlated with distance; therefore, we did not include ESD in our analysis. We modified the input code to fit the intercept model for each dataset (i.e., no covariates were included on the detection process). Doing so simplified direct comparisons among the different analytical frameworks.

#### 2.3.2 CDS approach

**Figure 2:**
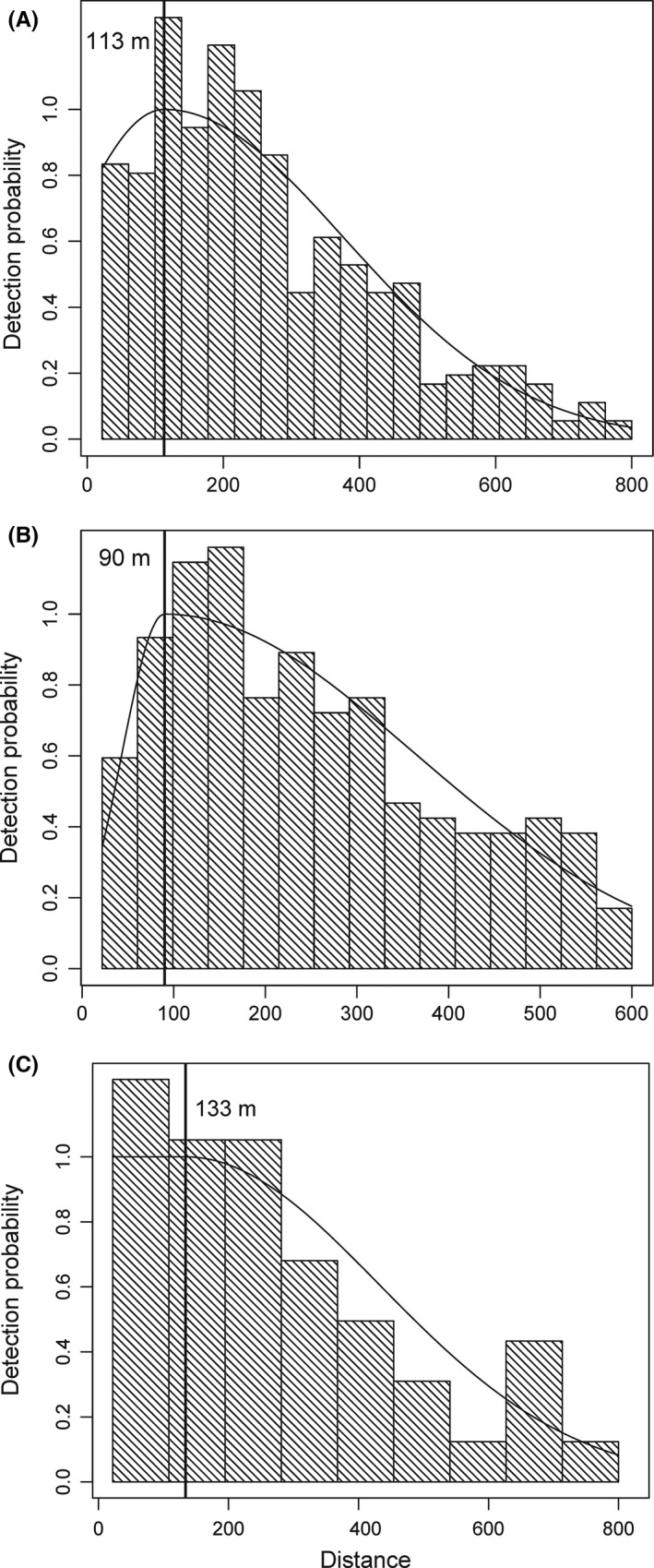
Histograms of detection distances and two‐piece normal detection functions for Katmai National Park and Preserve in 2004–2005 (A), Lake Clark National Park and Preserve in 2003 (B), and Katmai National Preserve in 2009 (C). The vertical line represents the estimated apex of the detection function based on the B‐C approach. The CDS approaches include only the data to the right of the vertical line

The B‐C approach generally requires much larger sample sizes (i.e., ≥150 detections; Walsh et al., 2010; Thompson, Peirce, & Mangipane, 2010) than a standard CDS approach (60–80 detections; Buckland et al., 2001) for model fitting. We suspected that fitting the mark–recapture model and the complicated detection function might be partially responsible for the larger sample size requirements because of the addition of several more model parameters. In addition, past results have shown that marginal detection probabilities at the apex of the detection function are quite high for both the pilot and the observer (Becker & Christ, 2015; Becker & Quang, 2009; Walsh et al., 2010), suggesting that joint *p*_d_ at the apex may approach 1.0 in many cases. To test these hypotheses, we created a dataset appropriate for a CDS analysis by left‐truncating each dataset at the apex of the composite detection curve estimated using the B‐C approach above, which left a monotonically declining subset of distances from that point (Figure 2). We also discarded the capture history information associated with each detection, which is the equivalent of the pilot and observer working together as a team to detect bear groups. We then fit each reduced dataset in a CDS framework (Buckland et al., 2001), assuming a half‐normal detection function and the basic Bayesian hierarchical model described by Schmidt et al. (2012) (see Supporting Information). We used the Gelman–Rubin diagnostic (Brooks & Gelman, 1998) and a visual inspection of the chains to assess convergence. For simplicity, we did not include covariates on the detection process, but instead relied on the “pooling robustness” characteristics of the CDS approach to unmodeled heterogeneity in detection (Buckland et al., 2001; Marques, Thomas, Fancy, & Buckland, 2007). Group size was assumed to be Poisson distributed, and we used minimally informative priors on all model parameters. Using this basic model structure, we then estimated the total number of bears within each study area. We compare these results to those based on the B‐C approach with respect to apparent bias and precision of the resulting estimators.

#### 2.3.3 Bayesian approach: Informed priors

To investigate the influence of sharing detection information among surveys, we used an informed prior on the scale parameter, σ, of the detection function to refit the less data‐rich LACL and KATM.PR datasets (Table 1) in the same CDS framework. For the prior in each analysis, we used the posterior distribution of σ from the CDS analysis of the KATM dataset, similar to what has been done in other distance sampling contexts (see Schmidt & Rattenbury, 2013). The remaining model parameters were given minimally informative priors. We compare these results to those of the previous analyses in the context of the bias‐variance tradeoff.

#### 2.3.4 Open‐distance model

We also suspected violation of the assumption that all bears were available to be sampled (Becker & Quang, 2009). Variation in factors such as viewing angle, coloration, or cover type may render some proportion of individuals “unavailable” for sampling during a particular survey of each transect (e.g., Wilson et al., 2014). Such incomplete availability can be considered to be a form of temporary emigration where an individual bear has effectively “emigrated” from the visible space at a particular point in time. The recent development of open‐distance sampling models (Sollmann et al., 2015) offered an opportunity to directly address this problem by allowing the population to be “open” to temporary emigration (i.e., variation in *p*_a_) between survey days. Essentially, during each survey day, a different proportion of the bears occurring in the survey area could be available for detection depending on factors such as survey conditions and bear behavior. Open‐distance models use variation among repeated surveys to estimate *p*_a_ and produce estimates of the entire population exposed to sampling over the survey period. Because the study areas were large relative to bear movement rates, *p*_p_ was presumed to be approximately 1.0. If closure was not maintained at the level of the study area (i.e., *p*_p_ < 1.0), then estimates of *p*_a_ would reflect the combination of incomplete *p*_p_ and *p*_a_.

In order to test the assumption of *p*_a_ ≈ 1.0, we selected a subset of the KATM data from 6 days when sampled transects were spatially distributed throughout the study area (Figure 1). This reduced dataset consisted of 168 detections on 323 transects, providing a set of six representative samples of the population in the study area for analysis. We fit a version of the temporary emigration open‐distance model described by Kery and Royle (2016) to these data within the framework of Schmidt et al. (2012). Under the open model, individuals are allowed to become temporarily unavailable for sampling during a particular survey day. Because the Schmidt et al. (2012) model is formulated to estimate the inclusion probability of individual groups within transects, rather than counts of groups within transects (see Supporting Information), we modeled temporary immigration as variation in the inclusion probability of bear groups among the six survey days. Because individual transects were not resampled through time, we conditioned inclusion probability on availability, which varied by date rather than individual transect. Because the transects were well distributed throughout the study area, we could then reasonably assume that the daily survey data adequately represented the study population available for sampling each day. Assuming population closure at the level of the study area, variation in detection among survey dates would then reflect variation in *p*_a_. In addition, under this formulation, incomplete detection of available bears at the apex of the detection function would be incorporated into the availability parameter. The resulting estimator of population size represents the superpopulation (i.e., Schwarz & Arnason, 1996) of bears exposed to sampling during the six sampling days, whereas estimators for the KATM data based on both the basic CDS and the B‐C approaches represent the population of bears available to be sampled throughout the duration of the survey (21 days total).

## 3 Results

**Figure 3:**
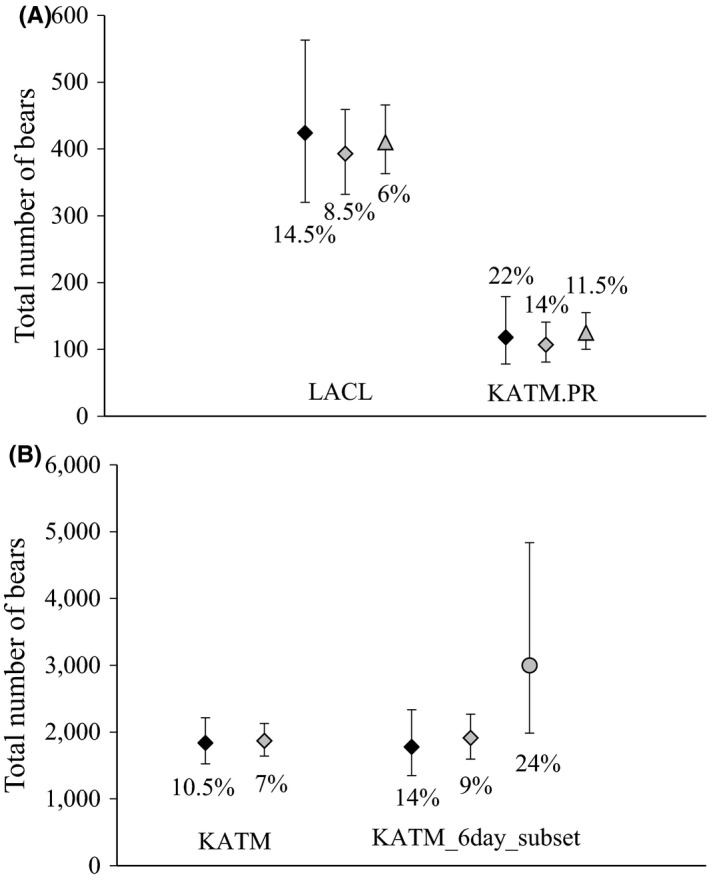
Comparison of estimates producing using the Becker‐Christ (B‐C) approach (black diamonds) versus the conventional distance sampling (CDS) approach (gray diamonds) for three datasets: Lake Clark National Park and Preserve in 2003 (LACL), the Katmai Preserve in 2009 (KATM.PR), and the entire Katmai National Park and Preserve in 2004–2005 (KATM). Estimates for LACL and KATM.PR from a Bayesian CDS analysis with an informed prior on sigma (scale parameter) based on the KATM results are represented by gray triangles. The estimates labeled KATM_6day_subset represent those based on a subset of the data including only days when survey coverage was representative. The gray circle in panel B represents the estimated superpopulation of bears exposed to sampling during the six survey days based on the open‐distance approach. Percentages represent approximate CVs for each estimate. Error bars are 95% CIs (B‐C) or 95% CrIs (CDS)

**Figure 4:**
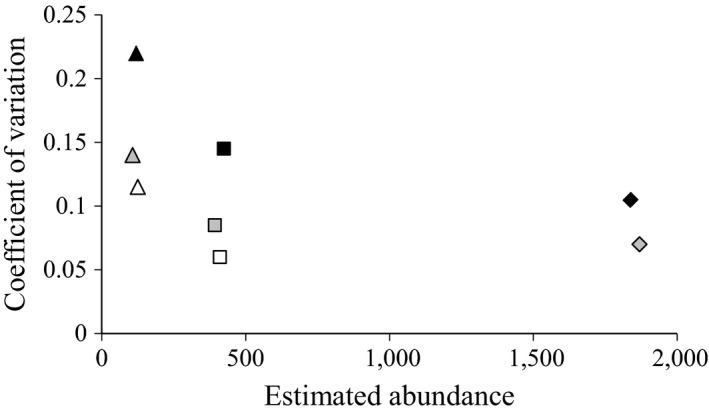
Comparison of the potential apparent bias (variation in estimated abundance) and estimator precision (represented as the coefficient of variation) for the Katmai Preserve in 2009 (triangles), Lake Clark National Park and Preserve in 2003 (squares), and the entire Katmai National Park and Preserve in 2004–2005 (diamonds) based on three different analytical approaches. Black symbols represent the Becker‐Christ approach, gray symbols represent the conventional distance sampling approach, and open symbols represent the conventional distance sampling approach incorporating an informed prior on the scale parameter of the detection function

The B‐C approach resulted in the least precise estimators of bear abundance for each of the three areas (Figure 3). Precision was especially low (i.e., CV = 22%) for the KATM.PR dataset where <50% of the recommended number of detections had been recorded. Estimators using the CDS approach were approximately 30–40% more precise (based on reduction in CVs) and exhibited little apparent bias (Figures 3 and 4). In comparing the point estimates between the two methods, we found little evidence that discarding the mark–recapture information in the CDS approach caused appreciable or consistent apparent bias relative to the B‐C results (i.e., −7%, −9%, +2% for the datasets from LACL, KATM.PR, and KATM, respectively; Figure 4). Error bars around estimates overlapped substantially in all cases. Precision was further increased using an informed prior on the scale parameter of the detection function, σ, based on the KATM results, for the LACL and KATM.PR CDS analyses, producing estimates 50–60% more precise than when analyzed independently under the B‐C approach (Figures 3 and 4). Apparent bias was negligible, suggesting that the detection process was very similar among the three survey areas.

Surprisingly, the results based on the open‐distance model showed that *p*_a_ was approximately 65% on average for the 6‐day KATM dataset, suggesting that *p*_a_ << 1.0, which in turn implied substantial underestimation of population abundance under the B‐C and CDS approaches (Figure 3). The resulting estimate from the open‐distance approach is interpreted as the superpopulation of individuals occurring within the study area that are exposed to sampling over the course of the survey, arguably the true population of interest. The precision of the superpopulation estimator was low (CV = 24%), likely because information from only 6 days was available to estimate the additional parameter for *p*_a_ in this example. Overall our combined results suggested that incomplete detection at the apex caused little apparent bias, whereas *p*_a_ << 1.0 can represent a large source of negative apparent bias under the standard MRDS design when unaddressed. Any apparent bias remaining in the CDS analyses caused by excluding the mark–recapture information was included in the estimates of *p*_a_ and would not have biased the resulting superpopulation estimators.

## 4 Discussion

This work was motivated by a desire to develop logistically feasible and cost‐efficient survey and analytical methodologies that would yield reasonably unbiased and precise estimators of abundance of brown bears in Alaska. The MRDS protocol is commonly employed when assessing bear populations in large landscapes; however, obtaining the recommended number of bear group detections for model fitting in the B‐C analytical framework is often cost prohibitive. This led us to reevaluate model complexity in the context of the assumptions inherent in the B‐C approach. We found that this well‐established and widely implemented approach for estimating bear abundance was suboptimal in the context of apparent bias and estimator efficiency. Our findings are especially important given the potential for long‐term reductions in cost and effort under simplified protocols (see “Step 10” in Reynolds, Knutson, Newman, Silverman, & Thompson, 2016). Although the B‐C approach formally accounted for the entirety of the complex detection process inherent in the bear survey data, the additional model complexity did not improve inference relative to much simpler approaches requiring estimation of many fewer parameters. A simpler CDS approach consistently produced estimators with higher precision and little apparent bias as compared to the B‐C approach, whereas the open‐distance model indicated a potentially severe violation of the assumption of *p*_a_ ≈ 1.0. These findings suggested that in the future aerial line‐transect surveys, the collection of mark–recapture information on individual groups is unnecessary, and these surveys should be redesigned to facilitate the estimation of *p*_a_. Any incomplete detection at the apex of the detection function would be directly incorporated into estimates of *p*_a_ under the open‐distance framework. Together these modifications would improve estimator efficiency and greatly simplify field efforts, making bear surveys of this type much more feasible to implement.

All survey designs require explicit assumptions about the detection process, although these assumptions can go unrecognized or ignored. A variety of survey techniques, including distance sampling approaches, address perception bias while generally assuming all individuals are present and available for sampling. These assumptions are critical, and their violation can result in improper inference (Nichols et al., 2009; Schmidt et al., 2013). In the context of long‐term monitoring of bear populations, these sources of bias can have important consequences for confounding trend estimators (Kery, Royle, & Schmid, 2005; Pollock et al., 2002). Violations of the assumption of *p*_a_ = 1.0 is known to cause large negative bias in a variety of settings (e.g., Bailey, MacKenzie, & Nichols, 2014; Efford & Dawson, 2012; Kendall & White, 2009; Wilson et al., 2014). In the case of occupancy models, such bias alters survey inference to some form of “use” (Mordecai, Mattsson, Tzilkowski, & Cooper, 2011; Schmidt, Flamme, & Walker, 2014), the utility of which must be carefully considered relative to survey objectives. Careful attention to the role of each component of the detection process is critical to proper inference and is an important consideration when designing bear surveys.

Field data on bear populations are generally expensive to collect, and sample size requirements play a large role in assessing whether a given survey approach is practical in the context of study objectives (e.g., Reynolds et al., 2011). Our work suggested that critical assessments of design assumptions and the application of new analytical tools can result in dramatic reductions in both the cost and effort involved with spatially extensive bear surveys, while also revealing hidden biases. We found that modification of model structure and revised treatment of the detection process increased precision by ≥50% using reduced versions of the same datasets, under the standard assumption of *p*_a_ ≈ 1.0. Based on these findings, future bear surveys could be simplified logistically (e.g., mark–recapture data need not be collected), and basic sample size requirements may be reduced. However, to address large apparent bias due to *p*_a_ < 1.0, design changes may also require some reallocation of effort. For example, future surveys may be improved by selecting transects from throughout the survey area each day so that incomplete availability can be assessed directly through the use of open‐distance approaches. Although simulations may be needed to identify appropriate sample sizes under an adjusted protocol, we expect that overall cost could be reduced substantially.

Estimator bias caused by imperfect detection continues to attract considerable attention from ecologists and biometricians, but estimator precision is an equally important consideration. As we have shown, increased model complexity can reduce precision; therefore, additional structure should be considered in terms of its effect on total error, not just bias alone. The shape of the histogram of detection distances and the presumption of a lack of complete detection at the apex led to the development of complex models of the detection process for bear survey data (i.e., Becker & Christ, 2015; Becker & Quang, 2009). These added components initially appeared reasonable; however, the simpler CDS approach substantially reduced total error despite a simplification of some assumptions. This highlights the importance of reassessing assumptions or considering alternative approaches as field and analytical techniques are developed through time in the context of their influence on total estimator error rather than focusing disproportionately on a single component of estimator quality.

Sample size demands are directly related to estimator precision, although the use of informed priors in a Bayesian framework may be advantageous (Gelman et al., 2004; King et al., 2010; Link & Barker, 2010). Despite the possible benefits of sharing information among surveys, the use of informed priors remains uncommon in wildlife surveys, possibly due to concerns that objectivity may be compromised (Morris, Vesk, McCarthy, Bunyavejchewin, & Baker, 2015). We found that sharing information on a common detection process dramatically increased the precision of our estimates of bear abundance with little effect on apparent bias. The use of informed priors on detection parameters has been found to have similar advantages in other distance sampling applications (i.e., Schmidt & Rattenbury, 2013) and would likely be useful for bear surveys in particular because the basic field methodology has been standardized and can be repeated in both time and space. Unless survey conditions are expected to be highly variable, we argue that the use of prior information on the detection process should be a default approach to increase the precision of estimators bear abundance collected using distance sampling protocols.

Assessing assumptions regarding each component of the detection process is often difficult without ancillary data. Recent developments of open population models for unmarked individuals (i.e., Dail & Madsen, 2011) provide a suite of tools that may be used to assess some of these assumptions directly as has been done in a mark–recapture framework (e.g., Kendall et al., 1997; Schwarz & Arnason, 1996). We used open‐distance models to assess *p*_a_ and found that a large proportion of bears were likely unavailable during the average survey day. We expect other similar applications (e.g., dynamic occupancy models) might prove equally useful in assessing assumptions for other survey types. When wildlife surveys are repeated over time (e.g., annually) for monitoring purposes, open population models may also be used to extract information on population dynamics (Dail & Madsen, 2011; Schmidt et al., 2015; Zipkin et al., 2014), further increasing their value for science and management. We expect this class of models will be useful in increasing the amount of available information on bear populations, as well as a variety of other species.

## Conflict of Interest

None declared.

## Data accessibility

All data used for the applied example will be made available at: https://irma.nps.gov/DataStore/.
