# Supplementary material for: Improving inference for aerial surveys of bears: The importance of assumptions and the cost of unnecessary complexity
Source: Ecol Evol. 2017 May 25;7(13):4812–21. doi: 10.1002/ece3.2912 (PMC5496527; doi:10.1002/ece3.2912)
Supplement: Supplementary file 1 [file ECE3-7-4812-s001.docx]

**Supporting Information**

OpenBUGS code representing the main model statements for the conventional distance sampling approach based on Schmidt et al. (2012) and the open distance sampling model based on a modification of the presentation by Kery and Royle (2016).

##Main model statement for the CDS model

for(i in 1:ntransects){

logit(psi1[i])<-psi2

for(j in 1:ngroups){ #includes many NA's (i.e. data augmentation)

w[i,j]~dbern(psi1[i]) #probability of occurrence

x[i,j]~dunif(0,5.11) #draw distances for unobserved groups

clust[i,j]~dpois(cmean[i,j]) #cluster size sub-model

cmean[i,j]<-clust.int

sigma1[i,j]<-sigma.int #scale parameter

sigma[i,j]<-exp(sigma1[i,j])

p[i,j]<-exp(-(x[i,j]*x[i,j])/(2*sigma[i,j]*sigma[i,j])) #detection function (half-normal)

mu[i,j]<-w[i,j]*p[i,j]

y[i,j]~dbern(mu[i,j])

}

}

##Main model statement for the Open Distance Sampling model

for(i in 1:ntransects){

logit(psi1[i])<-psi2

logit(phi[i])<-phi.int[date[i]]

psi1.2[i]<-psi1[i]*phi[i]

for(j in 1:ngroups){ #includes many NA's (i.e. data augmentation)

w2[i,j]~dbern(psi1[i]) #probability of being in superpopulation

w[i,j]~dbern(psi1.2[i]) #probability of occurrence given availability

x[i,j]~dunif(0,5.11) #draw distances for unobserved groups

clust[i,j]~dpois(cmean[i,j]) #cluster size sub-model

cmean[i,j]<-clust.int

sigma1[i,j]<-sigma.int #scale parameter

sigma[i,j]<-exp(sigma1[i,j])

p[i,j]<-exp(-(x[i,j]*x[i,j])/(2*sigma[i,j]*sigma[i,j])) #detection function (half-normal)

mu[i,j]<-w[i,j]*p[i,j]

y[i,j]~dbern(mu[i,j])

}

}
